# Interleukin-32 Gamma Stimulates Bone Formation by Increasing miR-29a in Osteoblastic Cells and Prevents the Development of Osteoporosis

**DOI:** 10.1038/srep40240

**Published:** 2017-01-12

**Authors:** Eun-Jin Lee, Sang-Min Kim, Bongkun Choi, Eun-Young Kim, Yeon-Ho Chung, Eun-Ju Lee, Bin Yoo, Chang-Keun Lee, Seokchan Hong, Beom-Jun Kim, Jung-Min Koh, Soo-Hyun Kim, Yong-Gil Kim, Eun-Ju Chang

**Affiliations:** 1 Department of Biomedical Sciences, University of Ulsan College of Medicine, Asan Medical Center, Seoul 05505, Korea; 2 Department of Rheumatology, University of Ulsan College of Medicine, Asan Medical Center, Seoul 05505, Korea; 3 Department of Endocrinology and Metabolism, University of Ulsan College of Medicine, Asan Medical Center, Seoul 05505, Korea; 4 Department of Biomedical Science and Technology, Konkuk University, Seoul 05066, Korea

## Abstract

Interleukin-32 gamma (IL-32γ) is a recently discovered cytokine that is elevated in inflamed tissues and contributes to pathogenic features of bone in human inflammatory rheumatic diseases. Nevertheless, the role of IL-32γ and its direct involvement in bone metabolism is unclear. We investigated the molecular mechanism of IL-32γ in bone remodeling and the hypothetical correlation between IL-32γ and disease activity in osteoporosis patients. Transgenic (TG) mice overexpressing human IL-32γ showed reduced bone loss with advancing age, increased bone formation, and high osteogenic capacity of osteoblast compared to wild-type (WT) mice through the upregulation of miR-29a, which caused a reduction of Dickkopf-1 (DKK1) expression. IL-32γ TG mice were protected against ovariectomy (OVX)induced osteoporosis compared with WT mice. Decreased plasma IL-32γ levels were associated with bone mineral density (BMD) in human patients linked to increased DKK1 levels. These results indicate that IL-32γ plays a protective role for bone loss, providing clinical evidence of a negative correlation between IL-32γ and DKK1 as bone metabolic markers.

Osteoporosis is a progressive bone disease that is caused by a dysfunction in bone remodeling, resulting in low bone mass and a consequent high risk of fractures^1^. Bone remodeling is maintained by a tight coupling of cellular activities by bone-resorbing osteoclasts (OCs) and bone-forming osteoblasts (OBs)^2^,^3^. Bone marrow-derived OC lineage cells induce the surface expression of receptor activator of nuclear factor-kappa B (NF-κB) (RANK) in response to macrophage-colony stimulating factor (M-CSF), which responds to RANK ligand (RANKL), leading to OC formation^3^. The recruitment of pre-OBs follows OC-mediated bone resorption^3^. Conversely, mature OBs secrete osteoprotegerin (OPG), also known as osteoclastogenesis inhibitory factor, which binds to RANK and blocks excessive OC formation^3^. This coupling of OB-mediated bone formation to bone resorption is impaired with aging and estrogen deficiency^4^, resulting in bone loss due to less bone formation than bone resorption^2^. In addition, bone loss is closely related to immunity^5^,^6^. Activated T cells can produce RANKL and other cytokines, and excessive OC activation is observed in osteoporosis^7^ and rheumatoid arthritis (RA)^8^. Pro-inflammatory cytokines, such as IL-1, IL-6, IL-17, TNFα, and IFNγ, enhance OC differentiation, but some of these can either inhibit or induce OB differentiation^9^,^10^,^11^,^12^, which contributes to the pathogenic features of bone in those diseases^7^,^8^. Thus, understanding the pathogenesis mediated by cytokines may provide new insight into therapeutic strategies to ameliorate bone loss.

Interleukin-32 (IL-32), originally called natural killer cell transcript 4, is a 27-kDa secretory glycoprotein^13^. IL-32 is mainly produced by T lymphocytes, natural killer cells, epithelial cells, blood monocytes, and fibroblast-like synoviocytes (FLS) in joints^14^,^15^. IL-32 is now recognized as an inflammatory cytokine that induces various other cytokines, such as IL-1β, TNF-α, IL-6, and IL-8^14^,^16^, and activates the p38MAPK and NF-κB signaling pathways in macrophages and T cells^14^. IL-32 has been studied in various clinical fields such as infectious diseases, autoimmune diseases (e.g., arthritis, psoriasis, ulcerativecolitis, Crohn’s disease), cancers, vascular disorders, and chronic obstructive pulmonary diseases^16^,^17^. IL-32 has transcriptional splice variants, encoding 6 different isoforms (IL-32α, IL-32β, IL-32γ, IL-32δ, IL-32ε, and IL-32ξ) with functional differences^18^. IL-32γ is the most active isoform among the IL-32 isoforms and has the same biological activity in mouse cells^13^. Thus, the physiological function of human IL-32γ has been explored in murine models of various diseases by incorporating the human IL-32γ gene in transgenic mice (IL-32γ TG)^19^. Accumulating evidence indicates that local elevation of IL-32γ in inflamed tissues is associated with the pathogenesis of inflammatory bone diseases, such as RA and ankylosing spondylitis (AS)^20^,^21^,^22^. IL-32γ stimulates OC formation *in vitro* in RA^20^,^21^,^23^ and actively enhances OB differentiation in AS^22^, indicating controversial effects on bone feature. These considerations led us to evaluate whether systemic IL-32γ displays an altered phenotype of bone metabolism and has the direct ability to promote bone formation under overexpression conditions of the human IL-32γ gene transgenic (TG) mice. We also found that IL-32γ TG resulted in the prevention of trabecular bone loss with aging and estrogen-deficiency. Interestingly, osteoporotic patients exhibited lower levels of human IL-32γ than healthy persons did, accompanied with higher Dickkopf-1 (DKK1) levels. These observations indicate systemic IL-32γ may be a bone-anabolic factor that can serve as a biomarker to represent a low risk of osteoporosis progression when coupled with DKK1.

## Results

### Increase in bone mass of IL-32γ TG mice with advancing age

**Figure 1:**
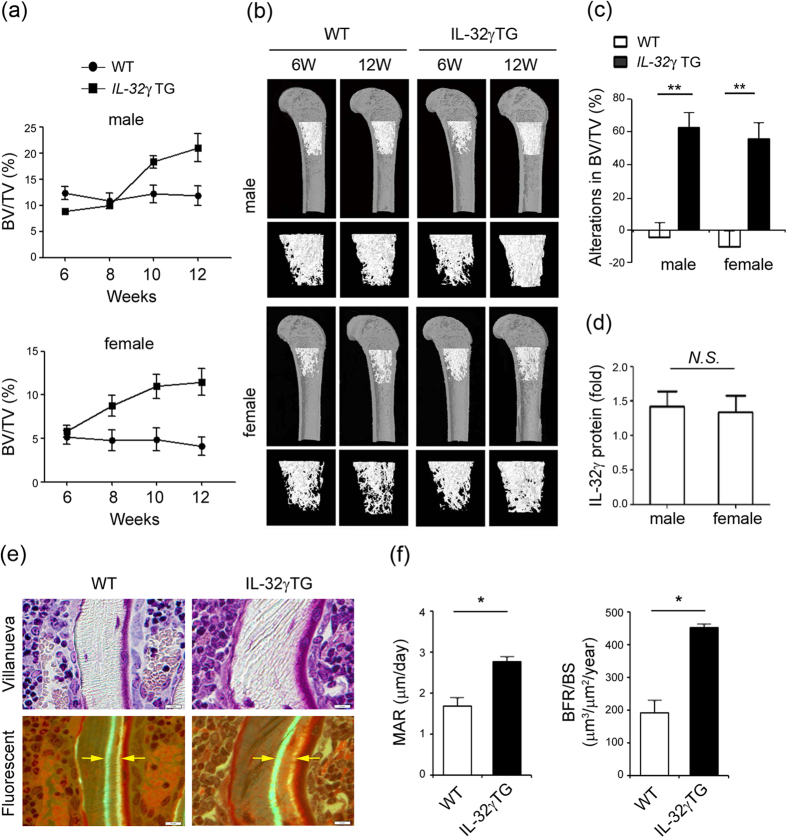
Overexpression of IL-32γ enhances bone volume and bone formation. (**a–c**) The femurs from WT and IL-32γ TG male and female mice were isolated at different ages (6, 8, 10, and 12 weeks) and fixed in 4% paraformaldehyde (PFA). The samples were examined by micro-CT imaging. The differences in bone phenotypes between WT and IL-32γ TG mice were analyzed. Bone volume per tissue volume (BV/TV, %) (**a**), micro-CT images of trabecular bone of femurs from WT and IL-32γ TG mice (**b**), and alterations (**c**) were calculated from femur sections using the micro-CT analysis program. (**d**) Serum IL-32γ levels in female and male TG mice were measured by enzyme-linked immunosorbent assay (ELISA). The results shown are the means ± standard deviation (SD) of 10 mice/group. NS, not significant; **p < 0.01, *p < 0.05. (**e,f**) Calcein double labeling showing bone formation in 11-week-old WT and IL-32γ TG mice. The mice were injected with calcein twice with an interval of 4 days and sacrificed 2 days after the second injection. The femur bones were embedded, sectioned, and evaluated by Villanueva staining **(e)**. The images were taken using a light microscope and fluorescent light microscope. Scale bar, 10 μm. Histological quantification of mineral apposition rate (MAR, μm/day) and bone formation rate (BFR/BS, μm^3^/μm^2^/year) in WT and IL-32γ TG mice **(f)**. The results shown are the means ± SD of 10 mice/group. *p < 0.05.

To determine whether IL-32γ affected bone metabolism, we overexpressed human IL-32γ under the control of an endogenous promoter to mimic the increased gene dosage of IL-32γ. A three-dimensional visualization of the femur area using micro-computed tomography (micro-CT) analysis revealed that the bone volume per tissue volume (BV/TV, %) decreased with age in both female and male wild-type (WT) mice (Fig. 1a and b). IL-32γ TG mice showed increased bone volume with aging and demonstrated increases in the volume of distal femoral bones by 56.3% in female and 63% in male mice, with marked decreases in bone loss at 12 weeks of age relative to that in WT mice (Fig. 1c). The male and female IL-32γ TG mice displayed similar IL-32γ serum levels (Fig. 1d). The vertebrae also revealed a high-bone-mass phenotype in IL-32γ TG mice (data not shown).

To gain more direct evidence for the role of IL-32γ in bone formation, we explored the bone formation rate over a 7-day period using dynamic histomorphometric analysis with calcein labeling^24^. Villanueva staining and the calcein-labeled bone histomorphometric analysis (Fig. 1e) showed that the basal level of mineral apposition rate (MAR), a parameter that reflects individual OB-mediated bone formation in 11-week-old IL-32γ TG mice (2.77 ± 0.10 μm per day), was 1.7-fold higher than that in WT mice (1.67 ± 0.21 μm per day) (Fig. 1f). Similarly, the bone formation rate (BFR/BS), a bone turnover marker, was 2.4-fold higher in IL-32γ TG mice (451.1 ± 11.3 μm^3^ per μm^2^ per year) than WT mice (191 ± 36.4 μm^3^ per μm^2^ per year). These observations suggest that a systemic overexpression of IL-32γ causes an increase in trabecular bone mass with a comparable increase in bone forming activity *in vivo*, resulting in an osteopetrotic phenotype.

### Effect of IL-32γ on OB and OC differentiation

**Figure 2:**
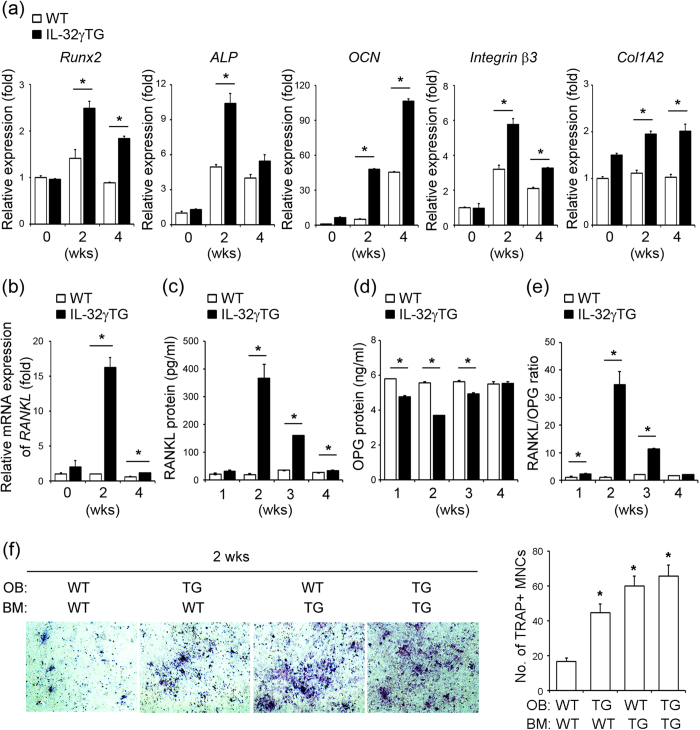
IL-32γ TG mice show increased expression of osteogenic markers in OB precursor cells. **(a,b)** Calvarial OB precursor cells were induced to differentiate for 2 and 4 weeks, and mRNAs were isolated. The expression of OB-specific genes, including runt-related transcription factor 2 (Runx2), alkaline phosphatase (ALP), osteocalcin (OCN), integrin β3, and collagen type 1 alpha 2 (Col1A2), was examined by qRT-PCR in differentiated OBs from WT and IL-32γ TG mice **(a)**. Expression of RANKL was determined by qRT-PCR in WT and IL-32γ TG mice **(b)**. **(c–e)** The amounts of RANKL **(c)** and OPG **(d)** secreted into the culture media from OBs of WT and IL-32γ TG mice at the indicated times were measured using ELISA kits, and the ratio of RANKL/OPG **(e)** was calculated. **(f)** Mouse bone marrow cells were co-cultured with calvarial osteoblastic precursor cells from WT and IL-32γ TG mice in the presence of 1α, 25(OH)_2_D_3_ (10^−8^ M) + PGE_2_ (10^−6^ M) for 5 days. The cells were then fixed and stained with TRAP. Representative images are shown in the left panel. The TRAP-positive (TRAP^+^) multinucleated cells (MNCs) containing three or more nuclei were counted under the light microscope. The quantitative data are expressed as means ± SD in the right panel. *p < 0.05 versus co-cultures from wild-type. Representative data of at least three independent experiments are shown.

To address the molecular mechanisms associated with enhanced bone forming activity in IL-32γ TG mice, we analyzed their capacity to regulate the genes affecting OB and OC differentiation in OBs. Calvarial OB precursor cells isolated from WT and IL-32γ TG mice were cultured in osteogenic media for 2 and 4 weeks. The expression of the typical OB-specific genes, including runt-related transcription factor 2 (Runx2), alkaline phosphatase (ALP), osteocalcin (OCN), integrin β3, and collagen type I alpha 2 (Col1A2)^25^, was determined. Runx2 is the essential transcriptional factor controlling OB differentiation that induces ALP activity for matrix maturation in the early stage of OB differentiation^25^. In addition, integrin β3 is a surface receptor of OBs, mediating adhesion to the collagen matrix^26^. OCN and Col1A2 are critical for proper mineralization of the bone and are specific markers for bone matrix synthesis^26^. Quantitative real-time polymerase chain reaction (qRT-PCR) analysis revealed that mRNA expression of Runx2, ALP, and integrin β3 significantly increased at 2 weeks in OB cultures from IL-32γ TG mice compared to those from WT mice, and gradually decreased at 4 weeks of OB cell culture (Fig. 2a). The expression of the OCN and Col1A2 markedly increased until 4 weeks of OB cell culture from IL-32γ TG mice (Fig. 2a). These results clearly demonstrated that OB differentiation gene expression was dramatically up-regulated in OBs from IL-32γ TG mice. Gene expression of RANKL and OPG, which are involved in OC formation, were also analyzed in OB cells from both WT and IL-32γ TG mice. Interestingly, the mRNA expression of RANKL was significantly higher in OBs from IL-32γ TG mice compared with that of WT mice (Fig. 2b). Secreted RANKL protein also markedly increased in IL-32γ TG mice compared with that in WT mice at 2~4 weeks of cell culture (Fig. 2c), whereas statistically less OPG protein was released in IL-32γ TG mice at 1~3 weeks of cultures (Fig. 2d). As a result, the ratio of RANKL protein to OPG protein was markedly increased in IL-32γ TG mice at 1~3 weeks of cultures (Fig. 2e). Given that RANKL is an essential osteoclastogenic factor^3^, we tested whether the capacity to increase RANKL production in cells from IL-32γ TG mice correlates with OC formation by co-culturing primary bone marrow cells with calvarial OB precursor cells in the presence of 1α,25(OH)_2_D_3_, the active form of vitamin D3, and prostaglandin E2 PGE_2_. This co-culture system provides a simplified version of the physiological bone microenvironment, in which OB and OC can participate in cross-talk with each other. Co-cultures from IL-32γ TG mice promoted the formation of tartrate-resistant acid phosphatase (TRAP)^+^ multinucleated cells compared with those from WT mice (Fig. 2f). Thus, the osteopetrotic phenotype observed in IL-32γ TG mice is due to increased OB differentiation, which is coupled to OC formation by increasing RANKL expression.

### Transcriptional regulation of the DKK1 gene by IL-32γ-dependent induction of miR-29a

**Figure 3:**
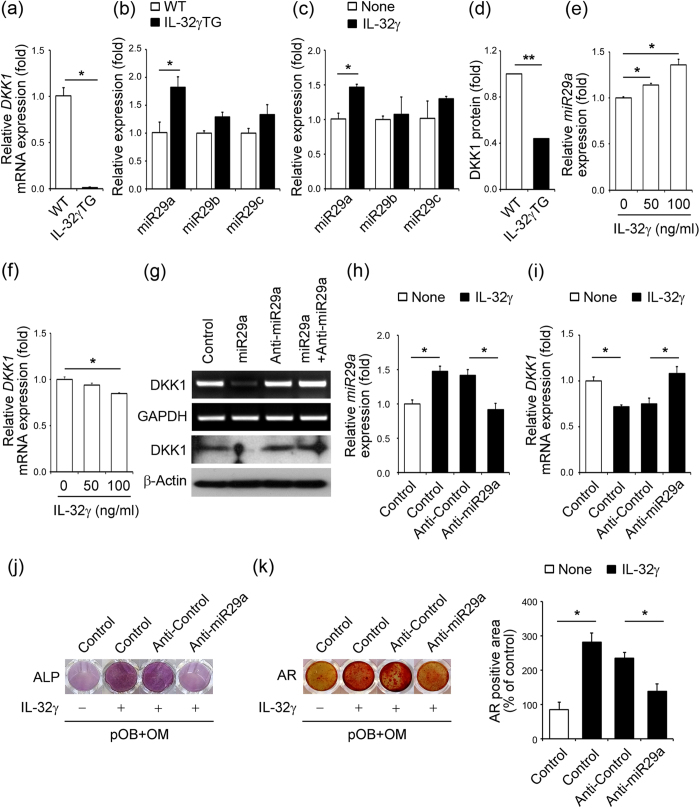
IL-32γ regulates OB differentiation through the induction of miR-29a to decrease DKK1 gene expression. **(a–d)** Calvarial OB precursor cells isolated from WT or IL-32γ TG mice were cultured in osteogenic medium. **(a)** The relative mRNA expression of DKK1 was evaluated in the cultured OBs from WT and IL-32γ TG mice. **(b)** The relative expression levels of miR-29a, miR-29b, and miR-29c were determined by qRT-PCR and normalized to U6 small RNA. **(c)** Calvarial OB precursor cells isolated from WT were cultured in osteogenic media in the presence of IL-32γ (100 ng/ml) for 24 hr. Relative quantities of miR-29 family were measured by qRT-PCR and normalized to U6 small RNA. **(d)** Culture supernatants of OB cells in WT or IL-32γ TG mice were collected and DKK1 protein levels were analyzed by ELISA. **(e,f)** Calvarial OB precursor cells isolated from WT were cultured in osteogenic media in the presence of IL-32γ (50 and 100 ng/ml) for 24 hr. The relative expression of miR-29a was measured by qRT-PCR and normalized to U6 small RNA **(e)**. The mRNA levels of DKK1 were analyzed by qRT-PCR **(f)**. **(g–i)** Primary osteoblastic cells were transfected with miR-29a and anti-miR-29a for 24 hr. The mRNA and protein levels of DKK1 were analyzed by RT-PCR and western blot. GAPDH and β-actin are internal controls. The full-length gels and blots are presented in Supplementary Fig. 1 **(g)**. Pre-osteoblastic cells (pOB) were transfected with control miRNA and anti-miR-29a, and cultured in osteogenic media with or without IL-32γ (100 ng/ml). Endogenous miR-29a expression was detected by qRT-PCR and normalized to U6 small RNA **(h)**. The mRNA expression of DKK1 was analyzed by qRT-PCR **(i)**. **(j)** ALP staining of OBs transfected with control, anti-control, and anti-miR-29a in the presence or absence of IL-32γ (100 ng/ml) was determined. **(k)** Alizarin red (AR) staining of OBs transfected with control, anti-control, and anti-miR-29a in the presence or absence of IL-32γ (100 ng/ml) was detected (left panel). The bar graph presents the AR-positive area measured in each cultured dish (right panel). The quantitative data are expressed as means ± SD. *p < 0.05.

A previous report showed that IL-32γ promotes osteogenesis via the suppression of DKK1, a negative regulator of Wnt/β-catenin-regulated osteoblastogenesis^22^, supporting a mechanistic explanation for the increased bone mass of IL-32γ TG mice. Indeed, the mRNA expression of DKK1 was significantly decreased in the primary calvarial OB precursor cells of IL-32γ TG mice relative to that of WT mice (Fig. 3a). We reasoned that the decreased transcription of DKK1 in OB precursor cells of IL-32γ TG mice might be due to direct regulation by miRNA, as in previous reports^27^,^28^. We thus investigated the potential involvement of IL-32γ–induced miRNAs in OB differentiation by regulating DKK1 levels in OB precursor cells. We searched for putative miRNAs targeting DKK1 among the family of miRNA involved in OB differentiation^29^ using a combination of TargetScan and miRanda. We selected the miR-29 family, including miR-29a, miR-29b and miR-29c, as candidate miRNAs. We determined whether ectopic expression of IL-32γ in TG mice or the administration of recombinant protein of IL-32γ to OB can induce the expression of the miR-29 family. Interestingly, the expression of miR-29a in primary OB precursor cells from IL-32γ TG mice was significantly higher than that in WT mice (Fig. 3b). However, no significant difference was observed in the levels of miR-29b and miR-29c in OB precursor cells between WT and IL-32γ TG mice (Fig. 3b). In addition, treatment of recombinant protein IL-32γ led to a significant increase in the expression of miR-29a, but not in miR-29b and miR-29c, in OB precursor cells (Fig. 3c). The protein level of DKK1 in serum from IL-32γ TG mice was much lower than that of WT mice (Fig. 3d). IL-32γ (50 ng/ml and 100 ng/ml) significantly increased miR-29a expression in OB precursor cells (Fig. 3e), whereas the mRNA expression of DKK1 was reduced in a dose-dependent manner (Fig. 3f). The forced expression of miR-29a inhibited the transcriptional and translational levels of DKK1 in OB precursor cells (Fig. 3g). The expression of miR-29a increased in OB precursor cells with IL-32γ treatment, and this was halted by transfection with a miR-29a inhibitor, anti-miR-29a, even in the presence of exogenous IL-32γ (Fig. 3h). The expression of DKK1 decreased with IL-32γ, which was restored by anti-miR-29a (Fig. 3i). The ectopic expression of anti-miR-29a inhibited rapid OB differentiation in response to exogenous IL-32γ treatment, as indicated by increased ALP activity (Fig. 3j) and the number of alizarin red (AR)-positive cells (Fig. 3k). These results demonstrate that IL-32γ hinders transcription of the DKK1 gene through the induction of miR-29a during OB differentiation.

### Effect of IL-32γ on ovariectomy-induced bone loss

**Figure 4:**
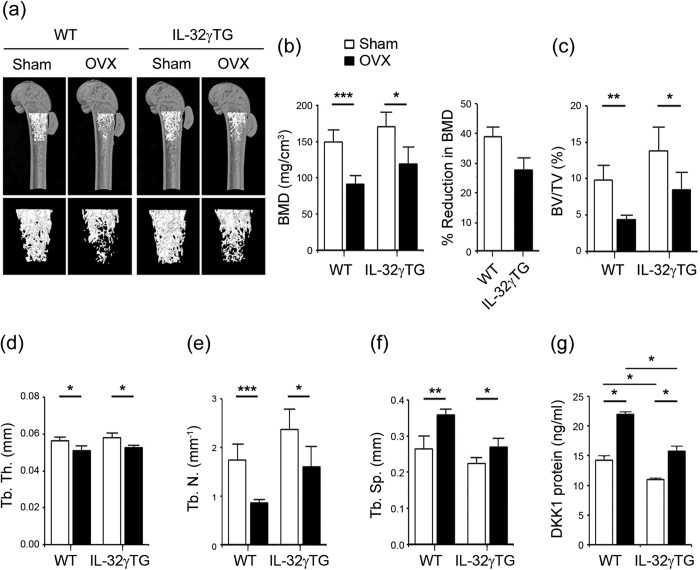
Overexpression of IL-32γ prevents bone loss induced by ovariectomy (OVX). (**a–f**) Wild-type and IL-32γ TG mice (8-week-old) were sham-operated or ovariectomized. After 4 weeks, the femurs from WT and IL-32γ TG mice were isolated and fixed in 4% PFA. The samples were examined by micro-CT imaging. Representative three-dimensional micro-CT images of femurs from WT and IL-32γ TG mice receiving either sham surgery or OVX (**a**). The histograms represent three-dimensional structural parameters of the femurs: micro-CT reconstruction of metaphyses of distal femurs, as well as bone mineral density (BMD) (**b**) in WT versus IL-32γ TG mice at 4 weeks after OVX. Three-dimensional morphometric analysis of bone parameters: bone volume per tissue volume (BV/TV) **(c)**, trabecular thickness (Tb. Th.) (**d**), trabecular number (Tb. N.) **(e)** and trabecular separation (Tb. Sp) (**f**). The results are the means ± SD of 6 mice/group. *p < 0.05, **p < 0.005, ***p < 0.001. (**g**) Wild-type and IL-32γ TG mice (8-week-old) were sham-operated or ovariectomized and sacrificed 4 weeks after OVX. The DKK1 protein level in the plasma from 12-week-old WT and IL-32γ TG mice were collected and determined by ELISA. Representative data from at least three independent experiments are shown. *p < 0.05.

To evaluate the consequences of IL-32γ overexpression on the development of osteoporotic condition *in vivo*, we analyzed bone phenotypes of female WT and IL-32γ TG mice after a sham- or ovariectomy (OVX)-operation. A three-dimensional visualization of the distal femoral area showed massive loss of trabecular bone following OVX in WT mice. In contrast, ovariectomized IL-32γ TG mice have significantly less reduction of bone loss compared to OVX in WT (Fig. 4a). Bone mineral densities (BMD, mg/cm^3^) were markedly reduced following OVX-operation in both WT and IL-32γ TG mice. The reduction in BMD was more than 38.81% in WT mice; BMD was less affected by OVX (27.53% of BMD) in IL-32γ TG mice (Fig. 4b). An analysis of the micro-CT data revealed that OVX significantly reduced bone parameters, including bone volume per tissue volume (BV/TV, %), trabecular thickness (Tb. Th., mm), and trabecular number (Tb. N., mm^–1^), whereas it increased trabecular separation (Tb. Sp., mm) in both WT and IL-32γ TG mice compared with sham-operated mice (Fig. 4c to f). The pattern of bone loss in vertebrae of WT and IL-32γ TG mice was almost consistent with that of femurs (data not shown). Accordingly, the induction rate of DKK1 protein levels in serum after OVX operation was lower in serum from IL-32γ TG mice than that of WT mice, with a dramatic increase in serum DKK1 in OVX-induced osteoporotic conditions (Fig. 4g), similar to a previous report^30^. These findings suggest that systemic overexpression of IL-32γ protects against OVX-induced bone loss, and that IL-32γ is closely related to DKK1 levels.

### Negative association of plasma IL-32γ and DKK1 in the development of osteoporosis

**Figure 5:**
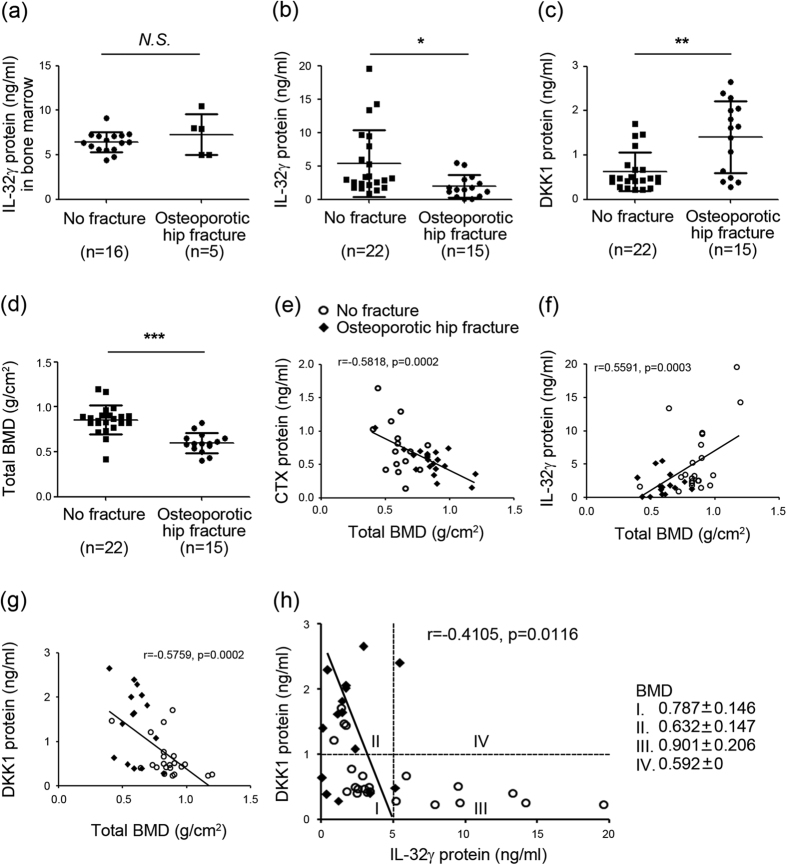
The decreased level of plasma IL-32γ is positively associated with bone mineral density but negatively related with DKK1 level in patients with osteoporosis. (**a**) Human IL-32γ levels in the bone marrow samples from patients with no fracture (n = 16) or osteoporotic hip fractures (n = 5) were measured using a commercially available ELISA kit. (**b,c**) Levels of human IL-32γ and DKK1 in the plasma samples of patients with no fracture (n = 22) and osteoporotic hip fracture (n = 15) were measured using a commercially available ELISA kit. Systemic IL-32γ levels (**b**) and DKK1 levels (**c**) in the plasma samples from patients with osteoporotic hip fracture or no fracture. (**d**) Comparison of BMD in total patients (n = 37). The bars show the means ± SD. NS, not significant; *** *p* < 0.001, **p < 0.01, *p < 0.05. (**e**) Concentrations of human CTX protein in the plasma were measured with an electrochemileuminescence immunoassay (ECLIA) kit. Relationship between plasma CTX (**e**), IL-32γ (**f**) or DKK1 levels (**g**) and BMD in total patients. (**h**) Correlation curve between IL-32γ and DKK1. The mean BMDs in total patients were categorized into 4 groups (I–IV) based on the cutoff values of DKK1 (1 ng/ml) and IL-32γ (5 ng/ml). Data are represented as the means ± SD.

We reasoned that lower BMD could be associated with decreased levels of IL-32γ because of the higher DKK1 levels in bone marrow fluid and peripheral blood from patients with osteoporosis^31^. We thus determined the levels of IL-32γ in the bone marrow and plasma of patients with osteoporotic hip fracture or no fracture. The clinical characteristics of human patients are listed in Table 1. The total number of hip fracture (HF) patients was 15, with 10 females (66.7%) and 5 males (33.3%). The 22 patients without HF consisted of 9 females (40.9%) and 13 males (59.1%). The mean ages for subjects with HF and without HF were 78.5 ± 8.7 years (range = 66–95 years) and 60.8 ± 13.4 years (range = 42–82 years), respectively (p < 0.0001). There were no significant differences in weight, height, and body mass index (BMI) between these two groups. Significant differences in BMD value (presented as Z-score) were observed at the femur neck, total femur, and trochanter. Despite evidence suggesting a role for local IL-32γ in the pathogenesis of inflammatory bone diseases^20^,^21^,^22^, there were no significant differences of the levels of bone marrow IL-32γ between patients with osteoporotic hip fracture (n = 5) or no fracture (n = 16) (Fig. 5a). The levels of plasma IL-32γ were significantly lower in osteoporotic hip fracture patients (n = 15) than in no fracture patients (n = 22) (Fig. 5b) (p < 0.05). These alterations were not affected by gender, age, or BMI (data not shown). Conversely, the plasma DKK1 levels were significantly higher in osteoporotic hip fracture patients than in no fracture patients (Fig. 5c) (p < 0.01). We next set out to correlate IL-32γ protein and DKK1 protein concentrations with BMD. As shown in Fig. 5d, the BMD was lower in osteoporotic hip fracture patients than in patients with no fractures. In this experimental cohort (n = 37), there was a strong negative correlation between BMD or plasma C-terminal telopeptide of type I collagen (CTX) protein concentrations (a known bone resorption marker) (*r* = −0.5818, *p* = 0.0002) (Fig. 5e). The plasma IL-32γ levels were positively associated with BMD (*r* = 0.5591, *p* = 0.0003) (Fig. 5f). In contrast, the plasma DKK1 levels show a negative correlation with BMD (*r* = −0.5759, *p* = 0.0002) (Fig. 5g). Subsequently, plasma IL-32γ levels were inversely associated with plasma DKK1 levels (*r* = −0.4105, *p* = 0.0116) (Fig. 5h). When patients were categorized into 4 groups according to the cutoff values of DKK1 (1 ng/ml) and IL-32γ (5 ng/ml), most patients in category III with a low DKK1 and high IL-32γ did not experience fracture and showed relatively higher BMD compared with those in category II (*p* = 0.003). Taken together, these observations indicate that systemic IL-32γ is negatively related with changes in DKK1 levels with the development of osteoporosis.

## Discussion

IL-32γ is induced by various inflammatory cytokines and activation of toll-like receptors (TLRs) in RA FLSs^32^, indicating that IL-32γ is an inflammatory cytokine^16^. Thus, it is common knowledge that IL-32γ may act as an enforcing factor in OC formation, like other inflammatory cytokines; it stimulates OC formation and inhibits OB differentiation following excessive bone erosion in inflammatory bone diseases^9^,^10^,^11^,^12^,^20^,^21^,^23^. In addition, IL-32γ synergistically enhances various cytokines, such as TNF-α and IL-1β, to further induce inflammation in local sites^16^. This makes conditions more favorable for osteoclastogenesis in RA joints by promoting OC formation and increasing RANKL in FLSs, contributing to inflammatory bone loss^20^,^23^. Conversely, the higher levels of IL-32γ in joints of ankylosing spondylitis relative to those of other inflammatory bone diseases are associated with abnormal bone formation and enhanced OB differentiation^22^. These contradictory reports emphasize that local IL-32γ may have a dual function in its effect on bone features, depending on the disease circumstances. The present data for IL-32γ TG mice shows an osteopetrotic phenotype with advancing age and increased bone forming activity *in vivo* (Fig. 1). IL-32γ potentiates both osteogenic and osteoclastogenic capacity of OBs by promoting OB differentiation and osteoclastogenic RANKL production (Fig. 2), supporting the idea that IL-32γ plays a role in the coupling of bone resorption to OB-mediated bone formation for maintenance of bone homeostasis.

Despite the clear protective function of IL-32γ in bone loss in this study, the underlying regulatory mechanism of IL-32γ alteration was not precisely determined. In fact, IL-32γ upregulation is linked to inflammatory cytokines in various cells^32^, thus, it is possible that IL-32γ could be increased in such condition, because osteoporosis is associated with increased inflammation^7^. In contrast, high levels of IL-32γ in mice during the process of bone remodeling observed in this study showed that IL-32γ could play a role in bone formation and maintenance of bone homeostasis with advancing age (Fig. 1) and following OVX operation (Fig. 4), which may indicate that IL-32γ regulation in bone metabolism is not linked to a phase in the inflammatory response. One possible explanation that has been suggested is the cross-talk signaling between IL-32γ and cyclooxygenase-2 (COX-2)^33^. COX-2 is an inducible rate-limiting enzyme in prostaglandin biosynthesis that is involved in RANKL production by OB^34^ and directly stimulates prostaglandin E2 (PGE_2_)-induced OC formation and further bone destruction^35^. Estrogen deficiency stimulates COX-2 expression, which correlates with osteoporosis in mice^36^. Thus, COX-2 inhibition prevents menopause-associated bone loss^37^, indicating the importance of COX-2 in OB during bone metabolism. Recent reports indicate that COX-2 upregulates IL-32γ production. Conversely, IL-32γ attenuates COX-2 expression and subsequent COX-2-derived PGE2 synthesis^33^. Therefore, it is probable that there is regulatory signaling for the cross-talk between IL-32γ and COX2 in OB after menopause; however, further investigation on this relationship is needed.

Emerging interest has been attributed to the involvement of microRNAs, which are small non-coding RNAs that regulate various gene expression patterns, including those related to bone formation/resorption-associated mechanisms^38^. For instance, several miRNAs were shown to inhibit the osteogenic inhibitor during stem cell differentiation into OCs^39^, regulate BMP-2 dependent OB differentiation and proliferation^40^, or contribute to the modulation of the Wnt signaling pathway^41^. In particular, miR-29b was found to promote OB differentiation by increasing bone matrix collagen type 1al expression^42^, mineralization reactions, and interaction with Wnt signaling pathway in OBs^41^. Ko’s group reported that miR-29a ameliorates glucocorticoid-induced bone loss^43^ and regulates excess glucocorticoid suppression of OB differentiation by regulating β-catenin acetylation via HDAC4^44^. miR-29a modulates the negative regulators of Wnt signaling, DKK1, Kremen2, and secreted frizzled related protein 2, which are direct targets of miR-29a^28^ and thereby osteogenic differentiation^28^,^44^. Our data showed that IL-32γ decreases DKK1 gene expression by upregulating miR-29a in OBs (Fig. 3), suggesting a molecular mechanism for the reduction of DKK1 by IL-32γ in OBs.

DKK1 is known to be a soluble inhibitor of Wnt/β-catenin signaling involving bone development and remodeling by affecting the bone microenvironment^45^. Forced overexpression of DKK1 in OB causes osteopenia by disruption of the hematopoietic stem cell niche and its function^45^. In addition, DKK1 activation inhibits OB differentiation from mesenchymal stem cells by inducing proliferation *in vitro*^45^, which causes estrogen-deficiency-mediated osteoporosis^30^,^45^. DKK1-deficient mice show high bone mass, and conversely, heterozygous overexpression of DKK in mice results in low bone mass^46^, suggesting a clinical application for anti-DKK1 antibody as a bone-anabolic agent to stimulate OB differentiation^46^,^47^. In this study, IL-32γ TG mice show a protective effect on bone loss, even in estrogen-deficiency conditions (Fig. 4). This is similar to the phenotype of DKK1 knockout mice in osteoporotic conditions^46^. These reports strongly support the mechanistic explanation of systemic IL-32γ and its functional link to DKK1. More importantly, since a sustained level of IL-32γ in TG mice inhibited DKK1 activation and consequently stimulated OB differentiation and retained bone mass (Fig. 1 and Fig. 2), we believe that IL-32γ may exhibit more potent bone-anabolic effects than bone-catabolic effects due to its potential to suppress DKK1 expression in OBs.

Given that high plasma DKK1 levels in osteoporosis patients correlate negatively with BMD^31^, our observation that the plasma level of IL-32γ (Fig. 5) is correlated with BMD with advancing age supports a notion of the negative correlation between systemic IL-32γ and DKK1 for maintenance of bone mass. Our finding showing no significant changes in the bone marrow levels of IL-32γ between osteoporotic hip fracture patients and no fracture patients (Fig. 5) also indicates that systemic IL-32γ may be responsible for the maintenance of bone metabolism, rather than local concentrations. However, there is insufficient information to know the physiological relevance of systemic levels of IL-32γ in pathogenesis. Even though the concentration of α-isoform is elevated in the blood of patients with chronic obstructive pulmonary disease^48^, myasthenia gravis^49^, and stomach cancer^50^, the clinical meaning of high blood levels of IL-32α in patients with various diseases is still obscure. In addition, the pathogenic deterioration seen in RA and AS cases is caused by abnormal local accumulation of IL-32γ in inflamed tissues, with no considerable difference between patients’ blood and that of a healthy population^20^,^21^,^22^. Nevertheless, decreasing the IL-32γ level in blood could give us information on decreased activity of bone regeneration in patients with osteoporosis. These observations highlight the distinction in the physiological significance of circulating IL-32γ and its local effect, suggesting that the plasma levels of IL-32γ and DKK1 can serve as biomarkers to predict the progression of osteoporosis. However, relatively small numbers of osteoporosis patients were included in this study; therefore, the clinical significance of systemic levels of IL-32γ as a bone metabolic marker in osteoporosis patients should be evaluated by further study with a larger number of patients.

## Methods

### Sample collection and enzyme-linked immunosorbent assay (ELISA)

All experiments using mice were performed in accordance with the relevant guidelines and regulations on the use of animals approved by the Institutional Animal Care and Use Committee of the Asan Biomedical Research Institute of the Asan Medical Center (2013-02008). All biologic samples from patients were obtained with the approval of the Asan Medical Center Institutional Review Board (S2013-1564-0001). All methods were performed following the relevant guidelines and regulations on the use of human samples. Informed consent was obtained from all patients. Plasma or bone marrow samples from patients who underwent hip surgery due to osteoporotic hip fracture (n = 15) or other causes with no fracture, (n = 22) such as osteoarthritis, avascular necrosis (AVN) of the femoral head, and hip dislocation, were collected as previously described^51^. All samples were collected at the Department of Orthopedic Surgery, Asan Medical Center, Seoul, Korea. We excluded patients who had taken drugs that could affect bone metabolism, patients with hyperthyroidism or rheumatoid arthritis, or patients with high-impact fractures, such as a motor vehicle accident or falling. Patient information was obtained during their diagnosis at the time of operation. Blood and bone marrow fluid samples were collected during hip surgery, centrifuged to exclude cell components, and stored in −80 °C until the assay. The concentrations of human IL-32γ were measured using commercially available kits obtained from YbdY (Seoul, South Korea). Commercial ELISA kits (R&D Systems, Minneapolis, MN) were used to determine the murine DKK1, RANKL, and OPG proteins. Human DKK1 levels were measured using an ELISA kit (Quantikine, R&D Systems, Minneapolis, MN) according to the manufacturer’s instructions. The C-terminal telopeptide of type I collagen (CTX) was measured by an electrochemiluminescence immunoassay (ECLIA) kit (Roche Diagnostics, Basel, Switzerland).

### Micro-computed tomography analysis

Distal femoral bones dissected from mice were fixed in 4% PFA and scanned by micro-computed tomography (micro-CT) using the Skyscan 1072 system (14.85 μm pixel size, 50 kVp, 200 μA, 0.5 mm AI filter, Skyscan, Kontich, Belgium). Datasets were reconstructed using modified cone beam reconstruction software (NRecon) with a Feldkamp-based algorithm and were segmented into binary images using adaptive thresholding. After the acquisition of 200 tomographic slices, a bone volume analysis was performed using CTan software (ver 1.6). Three-dimensional surface-rendered models were generated using CTan software and visualized using CTVol (Bruker-micro-CT).

### Bone mineral density (BMD) measurement

For BMD analysis in an ovariectomized mouse model, eight-week-old female WT and IL-32γ TG mice (n = 6 per group) were sham-operated or ovariectomized under anesthesia, and bone loss was assessed four weeks later^52^. We extracted the femurs and vertebras from euthanized mice, fixed them in 4% PFA, and subjected them to micro-CT analysis with the Skyscan 1072 microtomograph system as described above. BMD was measured in the region of interest with micro-CT (Skyscan). Areal BMD (g/cm^2^) in lumbar spine and proximal femur (femoral neck, total hip) was measured using dual-energy X-ray absorptiometry (DXA; QDR 4500 A, Hologic Inc., Waltham, MA, USA)^51^.

### Bone histomorphometric analysis

Calcein labeling was conducted to estimate the levels of new bone formation *in vivo*. Briefly, mice were intraperitoneally injected with calcein (30 mg/kg of body weight) in a 2% sodium bicarbonate solution on the 1st and 5th days prior to sacrifice. The tibia and femora were fixed in 70% ethanol, embedded in methyl methacrylate, and sectioned. Sections were stained with Villanueva stain, and pictures were taken under a natural light and fluorescence microscope. Analysis of the bone formation rate per bone surface was performed using BioquantOsteo (Bioquant Image Analysis Corp.). The mineral apposition rate (MAR, μm/day) is the distance between the midpoints of the two calcein labels divided by the time between the midpoints of the interval. The bone formation rate per bone surface (BFR/BS, μm^3^/μm^2^/year) is the volume of mineralized bone formed per unit time and per unit bone surface.

### Reverse transcription-polymerase chain reaction (RT-PCR) and quantitative real-time PCR (qRT-PCR) analysis

Total RNA was isolated from cells using TRIzol Reagent (Life Technology, Carlsbad, CA). One microgram of RNA was reverse-transcribed using SuperScript II reverse transcriptase (Life Technologies, Carlsbad, CA) according to manufacturer’s instructions. qRT-PCR was performed using a LightCycler 480 SYBR Green I-step Kit and the LightCyclerR 480 Instrument II Real-Time PCR System (Roche Applied Science, Mannheim) according to the manufacturer’s instructions. The resulting cDNA was amplified by PCR using the following primers: mouse Runx2 (runt-related transcription factor2), 5′-TTC AAC GAT CTG AGA TCT GTG GG-3′ (forward) and 5′-GGA TGA GGA ATG CGC CCT A-3′ (reverse); mouse ALP (alkaline phosphatase), 5′-CCA ACT CTT TTG TGC CAG AGA-3′ (forward) and 5′-GGC TAC ATT GTT GAG CTT TT-3′ (reverse); mouse OCN (osteoclacin), 5′-CTG ACC TCA CAG ATC CCA AGC-3′ (forward) and 5′-TGG TCT GAT AGC TCG TCA CAA G-3′ (reverse); mouse integrin β3, 5′-CCC CGA TGT AAC CTG AAG GAG-3′ (forward) and 5′-GAA GGG CAA TCC TCT GAG GG-3′ (reverse); mouse Col1A2 (collagen type 1 alpha 2), 5′-CAG GAT GCC CGA AAA TTA GGG-3′ (forward) and 5′-ACC ACG ATC ACC TCT GGG T-3′ (reverse); mouse RANKL, 5′-AGC CGA GAC TAC GGC AAG TA-3′ (forward) and 5′-AAA GTA CAG GAA CAG AGC GAT G-3′ (reverse); mouse DKK1 (Dickkopf-related protein 1), 5′-GAG GGG AAA TTG AGG AAA GC-3′ (forward) and 5′-GCA GGT GTG GAG CCT AGA AG-3′ (reverse); mouse GAPDH (glyceraldehyde 3-phosphate dehydrogenase), 5′-AGC CAC ATC GCTCAG ACA-3′ (forward) and 5′-GCC CAA TAC GAC CAA ATC C-3′ (reverse).

To assess the expression of microRNA, 2 μg of total RNA was reverse-transcribed using miScriptHiFlex Buffer from the miScript II RT Kit (Qiagen). qRT-PCR was performed using an miRNA-specific miScript Primer Assay and the miScript SYBR Green PCR Kit containing the miScript Universal Primer and QuantiTect SYBR Green PCR Master Mix (Qiagen). U6 small RNA was used for normalization.

### MicroRNA oligonucleotide transfection

Synthetic miR-29a sense (miScriptmiRNA Mimic) and antisense (miScriptmiRNA inhibitor) oligonucleotides and scramble controls were obtained from Qiagen (Qiagen, Valencia, CA). Primary calvarial OB precursor cells were incubated overnight and transfected with 100–200 nM miR-29a sense and antisense oligonucleotides or the scramble control by Lipofectamine 2000.

### Western blotting analysis

Primary OB precursor cells and transfected OB cells were cultured and washed with ice-cold PBS and lysed in modified RIPA buffer (50 mM Tris/HCl [pH 7.4], 1% Nonidet P40, 0.25% sodium deoxycholate, and 150 mM NaCl) containing protease and phosphatase inhibitors. Cell lysates were centrifuged at 10,000 *g* for 15 min, the supernatants were collected, and the proteins were resolved in 10–12% SDS-PAGE gels. Separated proteins were transferred to polyvinylidenedifluoride membranes (Bio-Rad, Hercules, CA), and were then blocked for 1 hr with 5% bovine serum albumin (BSA) solution (MP Biomedicals, Auckland, New Zealand) in Tris-buffered saline containing 0.1% Tween 20. The membrane was incubated overnight at 4 °C with anti-DKK1 (sc-25516, Santa-Cruz) and anti-β-actin (A5441, Sigma) as primary antibodies, washed, and then incubated for 1 hr at 25 °C with horseradish peroxidase-conjugated secondary antibody. Reactive proteins were visualized using a chemiluminescence system (Merck-Millipore, Darmatadt, Germany).

### Osteoblast and Osteoclast Differentiation

For OB differentiation, primary mouse OB precursor cells were isolated from the calvariae of 1-day-old mice by six routine sequential digestions with 0.1% collagenase (Gibco BRL, Gaithersburg, MD) and 0.2% dispase (Roche, Penzberg, Germany)^53^. The cells were seeded onto 48-well culture plates at a density of 2 × 10^4^ cells/well and cultured in osteogenic medium (α-MEM, 10% FBS, 10 mM β-glycerophosphate, and 50 mg/ml ascorbic acid) for 1 to 4 weeks. OB differentiation and mineralization were assessed by detecting alkaline phosphatase (ALP) activity or by staining with Alizarin Red (AR). For OC differentiation, the bone marrow cells from the hind limbs of WT and IL-32γ TG mice were freshly isolated and co-cultured with mouse calvarial osteoblastic precursor cells in osteoclastogenic co-culture medium (α-MEM containing 1α,25(OH)_2_D_3_ (10^−8^ M), and PGE_2_ (10^−6^ M)) for 4∼7 days. The medium was changed every 3 days. The cultures were fixed and stained for tartrate-resistant acid phosphatase (TRAP) to observe OC formations.

### Statistical analysis

The difference between two groups was calculated using the Mann-Whitney U-test or an unpaired Student’s *t*-test, and the differences among three groups were analyzed by one-way ANOVA. The relationships among parameters were tested using Spearman’s rank correlation coefficient. Statistical analyses were considered significant with p values < 0.05.

## Additional Information

**How to cite this article**: Lee, E.-J. *et al*. Interleukin-32 Gamma Stimulates Bone Formation by Increasing miR-29a in Osteoblastic Cells and Prevents the Development of Osteoporosis. *Sci. Rep.* **7**, 40240; doi: 10.1038/srep40240 (2017).

**Publisher's note:** Springer Nature remains neutral with regard to jurisdictional claims in published maps and institutional affiliations.

## Supplementary Material

Supplementary Figure 1

## Figures and Tables

**Table 1:** Baseline characteristic of patients with/without osteoporotic hip fracture.

Variable	Patients without HF (n = 22)	Patients with HF (n = 15)	*P*
Sex, no. (%)			0.129
Female	9 (40.9)	10 (66.7)	
Male	13 (59.1)	5 (33.3)	
Age (y)	60.8 ± 13.4	78.5 ± 8.7	<0.001
Weight (kg)	60.5 ± 10.9	54.8 ± 11.5	0.140
Height (cm)	159.1 ± 10.4	156.5 ± 7.8	0.397
Body mass index (kg/m^2^)	23.8 ± 2.4	22.2 ± 3.9	0.193
Bone mineral density			
Femur neck Z-score	0.259 ± 1.462	−0.640 ± 0.752	0.034
Total femur Z-score	0.145 ± 1.129	−0.993 ± 0.793	<0.001
Trochanter Z-score	−0.209 ± 1.119	−1.107 ± 0.819	0.007
Ward Z-score	−0.341 ± 1.462	−0.740 ± 0.835	0.299
Lumbar spine Z-score	−0.715 ± 1.232	−0.508 ± 1.740	0.713

Values are presented as the mean ± standard deviation. Bivariate comparisons between groups were performed using the Mann-Whitney U test for continuous variables. HF hip fracture.
