# Supplementary material for: Interleukin-32 Gamma Stimulates Bone Formation by Increasing miR-29a in Osteoblastic Cells and Prevents the Development of Osteoporosis
Source: Sci Rep. 2017 Jan 12;7:40240. doi: 10.1038/srep40240 (PMC5228062; doi:10.1038/srep40240)

Interleukin-32 Gamma Stimulates Bone Formation by Increasing miR-29a in Osteoblastic Cells and Prevents the Development of Osteoporosis

Eun-Jin Lee[1], Sang-Min Kim[1], Bongkun Choi[1], Eun-Young Kim[1], Yeon-Ho Chung[1], Eun-Ju Lee[2], Bin Yoo[2], Chang-Keun Lee[2], Seokchan Hong[2], Beom-Jun Kim[3], Jung-Min Koh[3], Soo-Hyun Kim[4], Yong-Gil Kim[2,*], and Eun-Ju Chang[1,*]

[1]Department of Biomedical Sciences, University of Ulsan College of Medicine, Asan Medical Center, Seoul 05505, Korea

[2]Department of Rheumatology, University of Ulsan College of Medicine, Asan Medical Center, Seoul 05505, Korea

[3]Department of Endocrinology and Metabolism, University of Ulsan College of Medicine, Asan Medical Center, Seoul 05505, Republic of Korea

[4]Department of Biomedical Science and Technology, Konkuk University, Seoul 05066, Korea

Running title: Role of IL-32γ in bone metabolism

***Corresponding author**: Department of Biomedical Sciences, University of Ulsan College of Medicine, Asan Medical Center, Seoul 05505, Korea. Dr. Eun-Ju Chang (Tel: +82-2-3010-4262; fax: +82-2-3010-4234; email: ejchang@amc.seoul.kr) or Department of Rheumatology, University of Ulsan College of Medicine, Asan Medical Center, Seoul 05505, Korea. Dr. Yong-Gil Kim (Tel: +82-2-3010-3279; fax: +82-2-3010-6969; email: bestmd2000@amc.seoul.kr)

Supplementary Figure 1. Primary osteoblastic cells were transfected with miR-29a and anti-miR-29a for 24 hr. The mRNA level of DKK1 (a) and GAPDH (b) was analyzed by RT-PCR. The protein level of DKK1 (c) and β–actin (d) was evaluated using western blot. GAPDH and β-actin were included as internal controls. The full-length gels and blots are shown as below.

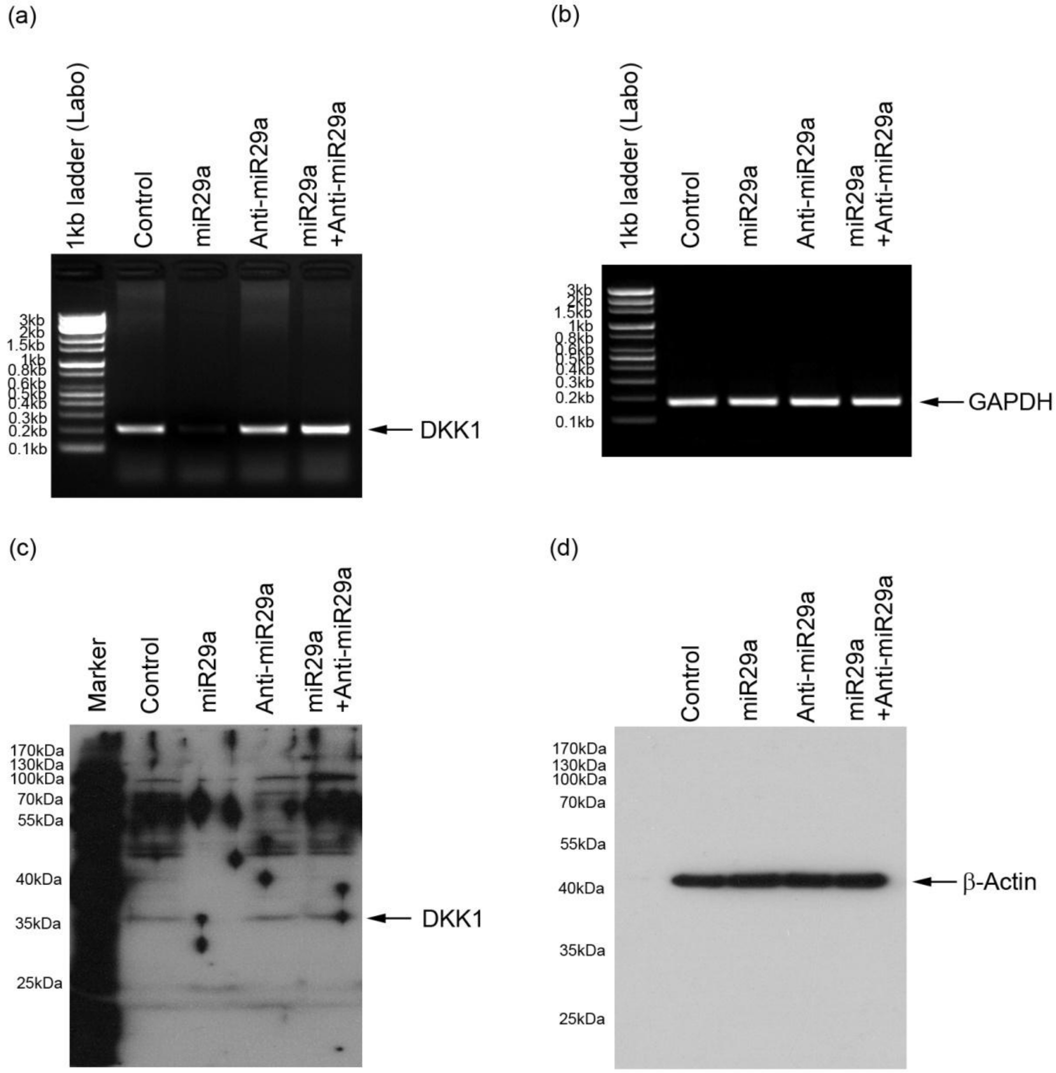

Supplement: Supplementary Figure 1 [file srep40240-s1.pdf]
